# Standardization of Aquafaba Production and Application in Vegan Mayonnaise Analogs

**DOI:** 10.3390/foods10091978

**Published:** 2021-08-24

**Authors:** Yue He, Sarah K. Purdy, Timothy J. Tse, Bunyamin Tar’an, Venkatesh Meda, Martin J. T. Reaney, Rana Mustafa

**Affiliations:** 1 Department of Chemical and Biological Engineering, University of Saskatchewan, Saskatoon, SK S7N 5A9, Canada; yuh885@mail.usask.ca (Y.H.); venkatesh.meda@usask.ca (V.M.); 2 Department of Plant Sciences, University of Saskatchewan, Saskatoon, SK S7N 5A8, Canada; sarah.purdy@usask.ca (S.K.P.); timothy.tse@usask.ca (T.J.T.); bunyamin.taran@usask.ca (B.T.); martin.reaney@usask.ca (M.J.T.R.); 3 Prairie Tide Diversified Inc., 102 Melville Street, Saskatoon, SK S7J 0R1, Canada; 4 Guangdong Saskatchewan Oilseed Joint Laboratory, Department of Food Science and Engineering, Jinan University, Guangzhou 510632, China

**Keywords:** aquafaba, chickpea, emulsifiers, egg replacement, mayonnaise

## Abstract

Canning or boiling pulse seeds in water produces a by-product solution, called “aquafaba”, that can be used as a plant-based emulsifier. One of the major problems facing the commercialization of aquafaba is inconsistency in quality and functionality. In this study, chickpea aquafaba production and drying methods were optimized to produce standardized aquafaba powder. Aquafaba samples, both freeze-dried and spray-dried, were used to make egg-free, vegan mayonnaise. Mayonnaise and analog physicochemical characteristics, microstructure, and stability were tested and compared to mayonnaise prepared using egg yolk. Chickpeas steeped in water at 4 °C for 16 h, followed by cooking at 75 kPa for 30 min at 116 °C, yielded aquafaba that produced the best emulsion qualities. Both lyophilization and spray drying to dehydrate aquafaba resulted in powders that retained their functionality following rehydration. Mayonnaise analogs made with aquafaba powder remained stable for 28 days of storage at 4 °C, although their droplet size was significantly higher than the reference sample made with egg yolk. These results show that aquafaba production can be standardized for optimal emulsion qualities, and dried aquafaba can mimic egg functions in food emulsions and has the potential to produce a wide range of eggless food products.

## 1. Introduction

Mayonnaise is a popular semisolid condiment that can improve the texture and flavour of foods such as salads, dips, and sandwiches. In recent years, because of health and environmental concerns, there has been an upward trend towards replacing egg with plant-based ingredients, especially in the formulation of mayonnaise analogs. Plant-based proteins [1,2], soymilk [3,4,5], starch, and modified starch [6] are reported to function as egg replacers that act as emulsifiers in mayonnaise analogs. To develop a vegan mayonnaise analog, one of the most difficult problems to solve is to create a stable emulsion structure that can withstand prolonged storage without coalescence or flocculation [7,8,9]. Emulsions are thermodynamically unstable systems, necessitating the use of emulsifiers to improve their storage stability [10].

Chickpea cooking water, commonly known as aquafaba, has recently been utilized as a vegan emulsifier in culinary formulations and as an egg replacement in vegan mayonnaise analogs. Aquafaba’s functional properties (emulsibility, foamability, gelation, and thickening properties) are attributed to its composition of protein, water-soluble/insoluble carbohydrates (oligosaccharide, starch, cellulose, hemicellulose, or lignin), polysaccharide-protein complexes, coacervates, saponins, and phenolic compounds [7,8,11,12,13,14,15]. Aquafaba is a by-product of pulse canning/boiling and freezing processes and hummus production. Using aquafaba in food products expands the market for plant-based foods, increases the demand for pulses, and reduces wastewater generated from some bean processes [7]. However, because most boiling and canning processes are designed to produce cooked pulse seed, the quality of aquafaba recovered varies significantly between manufacturers and within batches [16,17]. To assure aquafaba consistency and the quality of products made from it, standardization of aquafaba production is required. Different parameters, such as chickpea cultivars selected for aquafaba production and production conditions (i.e., water to seed ratio, temperature, pressure, time, and additives) should be addressed when standardizing aquafaba composition and functionality [16,18,19]. As aquafaba has a moisture content of more than 90% [16], it is also preferred to concentrate or dry aquafaba to improve transport efficiency by minimizing shipping costs, decreasing space required for storage, and preventing undesirable microbial growth [20]. To our knowledge, the impact of the drying process on the functional qualities of aquafaba has not yet been explored.

The purpose of this study is to standardize aquafaba production and drying processes, as well as to determine conditions that improve aquafaba powder’s function as an emulsifier in mayonnaise analog production. The emulsion properties of aquafaba produced from different cooking and drying methods were measured, and the physicochemical characteristics and stability of aquafaba-based mayonnaise analog and egg-based mayonnaise were examined and compared.

## 2. Materials and Methods

### 2.1. Materials

In our previous research, the Kabuli chickpea cultivar ‘CDC Leader’ was identified to produce a more favourable aquafaba with superior emulsion properties when compared with aquafaba produced from other cultivars [16]. Chickpea seeds (CDC Leader) were generously provided by Dr. Bunyamin Tar’an from the University of Saskatchewan, Crop Development Centre (CDC) (Saskatoon, SK, Canada). Chickpea seed was manually cleaned to remove broken seed, dust, and other foreign materials. Canola oil (purity 100%; ACH Food Companies, Inc., Terrace, IL, USA), eggs (Great Value large sized, Canada), baking soda (NaHCO_3_; Arm & Hammer by Church & Dwight Co., Inc, Mississauga, ON, Canada), and table salt (Windsor Salt, Pointe-Claire, QC, Canada) were purchased from a local supermarket (Walmart, Saskatoon, SK, Canada). Whole eggs were kept refrigerated at 4 °C, and before preparing mayonnaise, the yolk was separated from the egg white using an egg separator. White vinegar (No Name, Loblaws Inc., Toronto, ON, Canada) and sugar (Rogers granulated white sugar, Lantic Inc., Vancouver, BC, Canada) were purchased from a local supermarket (Real Canadian Superstore, Saskatoon, SK, Canada). Sodium dodecyl sulphate (SDS) was purchased from GE Healthcare (Mississauga, ON, Canada), and Nile red pigment was supplied by Sigma-Aldrich (Oakville, ON, Canada).

### 2.2. Aquafaba Production and Drying Method Standardization

Our goal was to identify the best conditions to produce aquafaba with superior emulsion properties. Tests were conducted in sequence by selecting the top-performing parameters for the next test. First, five aquafaba production conditions were evaluated, and the best aquafaba production conditions were selected based on the emulsifying activity index (EAI) and stability (ES). Second, aquafaba made with the optimized production methods was dried using five different drying methods. To make aquafaba powder, the drying methods that maintained aquafaba functionality were chosen. Third, the resulting aquafaba powder was used as an emulsifier to make analogs for mayonnaise.

#### 2.2.1. Optimization of Aquafaba Production

Dry chickpea seed (approx. 100 g) was washed and rehydrated by soaking in distilled water at a ratio of 1:4 (*w*/*w*) over time intervals of 1 and 16 h, at temperatures of 4 and 85 °C (Table 1). The soaking water was then discarded. The soaked chickpea seed (100 g) was rinsed with distilled water and combined with 100 mL distilled water with and without 0.2% NaHCO_3_ in 250 mL sealed glass jars and cooked for different times (20, 30, or 60 min) in a pressure cooker (70–80 kPa, and 115–118 °C; Instant Pot^®^ 7-in-1 multi-use programmable pressure cooker, IP-DUO60 V2, 6 quart/litres, Ottawa, ON, Canada). After cooking, the jars were cooled to room temperature (21 ± 1 °C) for 24 h. Cooled aquafaba was separated from cooked chickpea seed using a stainless-steel strainer and stored in a freezer (−18 °C) until use. Frozen aquafaba was thawed at 4 °C overnight and then warmed to 22 °C for 2 h before use. The aquafaba EAI and ES were measured according to He et al. [16].

#### 2.2.2. Comparison of Drying Methods

Liquid aquafaba sample (750 g) prepared from chickpea seed soaked in 4 °C water for 16 h then cooked for 30 min (condition B, Table 1) was divided into five equal parts (150 g) and dried using five different drying methods: freeze drying, spray drying, oven drying, Rotovap drying, and vacuum pressure drying. Freeze drying was performed by freezing the samples at −20 °C followed by drying in a FreeZone 12 Liter Console Freeze Dryer with Stoppering Tray Dryer (Labconco Corporation, Kansas City, MO, USA) until the sample temperature rose to −5 °C, indicating the sample had been thoroughly dried. Spray drying was completed at 150 °C, using a Büchi Mini Spray Dryer B-290 (Labortechnik AG, Flawil, Switzerland). Oven-dried samples were treated at 80 °C, using a VWR^®^ Signature™ Forced Air Safety Oven (Radnor, PA, USA) until they reached a consistent weight. Samples for Rotovap drying were placed in round-bottom flasks connected to a rotary evaporator (Büchi^®^ Model R-210 BUCHI Labortechnik AG, Switzerland) at 50 °C under vacuum pressure (50–100 mbar). Vacuum-dried samples were heated in a Fisherbrand™ Isotemp™ Model 281A vacuum oven (Fisher Scientific International, Ottawa, ON, Canada) at 60 °C for 12 h, under vacuum (33 mbar).

The residual moisture content of dried aquafaba samples was determined following the American Association of Cereal Chemists (AACC) method 44-15.02 [21]. Aquafaba powder was mixed with water to obtain the same concentration of solid materials as fresh aquafaba [16], and rehydrated aquafaba samples were mixed with canola oil to evaluate aquafaba emulsion properties.

### 2.3. Aquafaba Water Holding Capacity and Oil Absorption Capacity

Water holding capacity (WHC) and oil absorption capacity (OAC) of aquafaba powder were determined using the method described by Damian et al. (2018) [12] with minor modification. Aquafaba powder (1 g) was mixed with 20 g of distilled water and stirred for 1 min. The solution was then centrifuged for 10 min at 1860× *g*. After centrifugation, the supernatant was discarded, and the pellet weight was recorded. The WHC values were calculated as a ratio of the pellet weight to sample weight and were expressed as g water/g pulse cooking water (PCW). For OAC, distilled water was replaced with canola oil, and values were expressed as g oil/g PCW.

### 2.4. Development of Aquafaba Mayonnaise Analogs

Two formulations of egg-free analogs for mayonnaise were prepared using freeze-dried aquafaba and spray-dried aquafaba (analog A and B, respectively). Traditional mayonnaise, made with egg yolk, was used as a reference (mayonnaise C). The mayonnaise and analog formulations were modified from Raikos et al. (2019) [8] and included 80 mL canola oil, 4 mL vinegar (4% acidity), 0.5 g salt, 0.5 g sugar, and 15 g emulsifying agent. Canola oil was slowly added to the aqueous mixture (aquafaba/egg yolk, vinegar, sugar, and salt) and mixed for 5 min using a Kitchen Aid Ultra Power Mixer with a 4.3 L stationary bowl (Kitchen Aid, St. Joseph’s, MI, USA). Mayonnaise and mayonnaise analog samples were aliquoted and stored in a refrigerator (4 °C) for further analysis.

#### 2.4.1. Colour and pH

Mayonnaise and analog pH values were measured using a portable food and dairy pH meter (Hanna Instruments Ltd., Leighton Buzzard, UK). The colour characteristics were assessed using a Hunter Lab ColorFlex spectrophotometer (Hunter Associates Laboratory, Inc., Reston, VA, USA). Mayonnaise and analog colour, represented by lightness (L*), redness/greenness (±a*), and yellowness/blueness (±b*), was also determined initially after preparation and after 28 days of cold storage (4 °C). Chroma (*Ch*), colour difference from the control ($\Delta E_{1}{}^{*}$), and total colour change ($\Delta E_{2}{}^{*}$) of mayonnaise and analog samples during cold storage, were calculated using the following equations [8,18]:

(1) $$Ch=\sqrt{a^{*2}+b^{*2}}$$

(2) $$\Delta E^{*}=\sqrt{{\left(\Delta L^{*}\right)}^{2}+{\left(\Delta a^{*}\right)}^{2}+{\left(\Delta b^{*}\right)}^{2}}$$

#### 2.4.2. Mayonnaise and Analog Stability Test

Mayonnaise and analog samples (*F*_0_ = 10 g) were transferred to 15 mL centrifuge tubes and centrifuged for 30 min at 1860× *g*. The weight of the emulsified fractions (upper layer, *F*_1_) was measured after centrifugation, and emulsion stability (*ES*, %) was determined by Equation (3):

(3) $$ES=\left(\frac{F_{1}}{F_{0}}\right)\times 100\%$$

To measure heat stability, mayonnaise and analog samples were stored in an 80 °C water bath for 30 min before centrifugation. The heat stability was then characterized using Equation (3).

#### 2.4.3. Confocal Laser Scanning Microscopy

Mayonnaise and analog microstructure were analyzed with a Nikon C2 confocal laser scanning microscope (CLSM) (Nikon, Mississauga, ON, Canada) using a 543 nm laser with a 60× Plan-Apochromat VC (numerical aperture 1.4) oil immersion objective lens and five times digital zoom. The oil phase was stained using Nile red dye (0.01 wt.%). A drop of emulsion was placed on a microscope slide (Fisher Scientific, Nepean, ON, Canada) with a glass rod, covered with a coverslip (VWR International, Edmonton, AB, Canada) and observed under the CLSM.

#### 2.4.4. Droplet Size Distribution

Droplet size distribution of mayonnaise and analog samples was measured as a function of time (0, 7, 14, 21, and 28 days) using a static laser diffraction particle analyzer (Mastersizer 2000, Malvern Instrument, Montreal, QC, Canada) with a Hydro 2000S sample dispersion unit (containing water). The dispersion refractive index was 1.33, and the refractive index used for canola oil droplets was 1.47. Drops of samples were added to the sample dispersion unit until the obscuration index reached approximately 15%, and the average droplet size was reported in terms of volume-weighted mean diameter, *d*_43_, defined by Equation (4):

(4) $$d_{43}=\frac{\sum n_{i}d_{i}^{4}}{\sum n_{i}d_{i}^{3}}$$

where *d_i_* is the diameter of a droplet and *n_i_* is the number of droplets with the size of *d_i_*.

### 2.5. Statistical Analysis

Experiments were conducted in triplicate and the data were presented as means ± standard deviation (SD). Graphical illustrations were processed with Microsoft Excel^®^ 2018. Statistical analyses were completed using the Statistical Package for the Social Science (SPSS) version 25.0 (IBM Corp., Armonk, NY, USA). Analysis of variance (ANOVA) and Tukey’s post hoc statistical tests were used to evaluate significant differences in aquafaba physicochemical properties and mayonnaise and analog characteristics. Statistical significance was accepted at *p* < 0.05.

## 3. Results and Discussion

### 3.1. Optimization of Aquafaba Production

**Figure 1:**
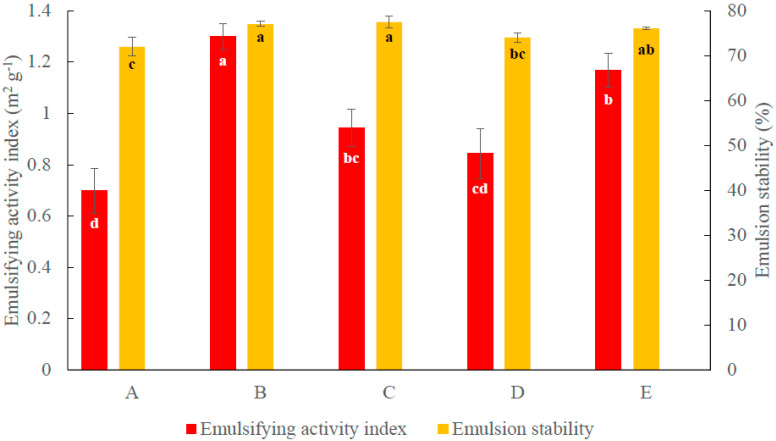
Emulsifying activity index and emulsion stability of aquafaba using different cooking conditions. A: Soaking seed in 4 °C water for 16 h then cooking for 20 min; B: soaking seed in 4 °C water for 16 h then cooking for 30 min; C: soaking seed in 4 °C water for 16 h and cooking for 60 min; D: soaking seed in 85 °C water for 1 h and cooking for 30 min; and E: soaking seed in 85 °C water with 0.2% (*w*/*w*) NaHCO_3_ for 1 h and cooking for 20 min. Different letters (a–d) refer to significant differences among different aquafaba production conditions according to Tukey’s test (*p* < 0.05).

The effects of soaking conditions (1 h at 85 °C; 16 h at 4 °C), cooking time (20, 30, and 60 min), and additive (NaHCO_3_) on aquafaba EAI and ES are shown in Figure 1. The highest EAI (1.30 ± 0.05 m^2^g^−1^) (*p* > 0.05) was obtained from aquafaba prepared by soaking chickpea seed in 4 °C water for 16 h and cooking for 30 min without additives (condition B, Table 1). The EAI dropped by 27% and 46% when the cooking time increased to 60 min or decreased to 20 min without additives (conditions C and A, 0.944 ± 0.072 m^2^g^−1^ and 0.699 ± 0.087 m^2^g^−1^, respectively). Soaking chickpea seed in 85 °C water for 1 h and cooking for 30 min (condition D, Table 1) saved soaking time but reduced the EAI of aquafaba to 0.843 ± 0.099 m^2^ g^−1^. By contrast, adding 0.2% (*w*/*w*) NaHCO_3_ to the soaking water (85 °C) (condition E, Table 1) slightly improved the EAI (1.17 ± 0.06 m^2^g^−1^); the EAI value under these conditions remained significantly lower than that of aquafaba prepared under condition B (*p* > 0.05). Aquafaba prepared under long cooking time conditions (conditions B and C) had comparable emulsion stability (77.1 ± 0.5% and 77.5 ± 1.4%, respectively), indicating that no significant difference was observed when prolonging the cooking time from 30 min to 60 min. However, decreasing the cooking time to 20 min (condition A) slightly decreased the aquafaba emulsion stability to 72.0 ± 2.1%.

The seed component hydration capacity was correlated with soaking time and temperature. During the soaking and cooking process, the outer cell layers of the seed coat transform into a selective membrane that regulates chemical diffusion to the cooking water (aquafaba). Sodium bicarbonate softens the seed coat and cotyledons and increases the concentration of compounds extracted from the seed into the cooking water [7]. Aquafaba emulsion properties are correlated to the concentration of protein, water-soluble/insoluble carbohydrates, polysaccharide–protein complexes, coacervates, saponins, and phenolic compounds [7,22,23]. Proteins in aquafaba, for example, are amphiphilic molecules with a low molecular weight (25 kDa) [17]. These molecules can aggregate at the water–oil interface, lowering the interfacial tension of the solution and forming an intermolecular cohesive film with enough elasticity to stabilize emulsions [7]. Polysaccharides enhance emulsion stability by gelling or changing the viscosity of the aqueous continuous phase, resulting in fewer droplet collisions. Previous research has also shown that the emulsion capacity of aquafaba from various pulses is proportional to their saponin and phenolic compounds concentration. These compounds bind with proteins and polysaccharides, changing their solubility and emulsifying properties [8,23]. During prolonged cooking times, heat and water damage the cell walls in the selective membrane layers, allowing larger molecular compounds to transfer into the aquafaba, lowering its emulsion properties [7]. On the other hand, short cooking time does not sufficiently soften chickpea seed to accelerate the leaching of compounds, limiting aquafaba solid material concentration and emulsion properties.

### 3.2. Comparison of Drying Methods

**Figure 2:**
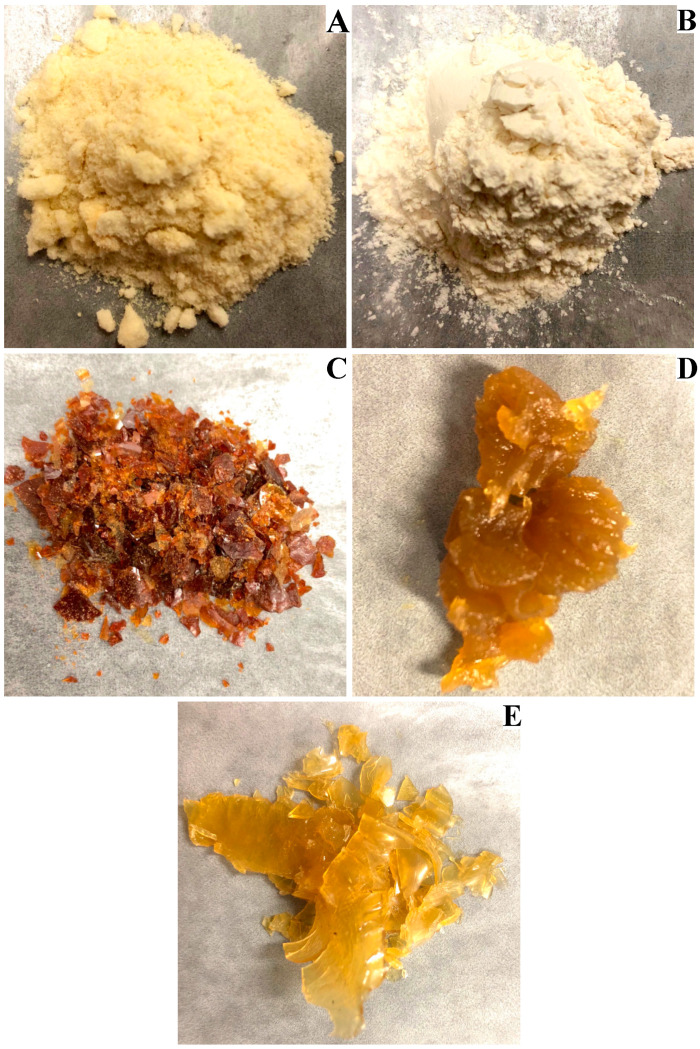
Aquafaba samples prepared by different drying methods: (**A**) freeze drying; (**B**) spray drying; (**C**) oven drying; (**D**) Rotovap drying; and (**E**) vacuum drying.

Given that aquafaba produced from chickpea ‘CDC Leader’ seed soaked in 4 °C water for 16 h then cooked for 30 min (condition B) had the highest EAI compared with other conditions, we chose aquafaba production condition B for further experiments. We investigated different drying methods in terms of drying time and aquafaba sensory and functional properties. Images of dried aquafaba powder prepared by different drying methods are provided in Figure 2. Freeze drying and spray drying methods resulted in a bright white and pale-yellow powder, respectively (Figure 2A,B). On the other hand, oven-dried aquafaba (Figure 2C) changed colour from pale yellow to dark brown, and its texture became brittle. Meanwhile, aquafaba dried via Rotovap drying (Figure 2D) resulted in a thick rubbery gel that adhered to the evaporator flask. Drying aquafaba using vacuum drying was slow and resulted in a rubbery sheet that had a higher moisture content compared with aquafaba dried using other methods (Figure 2E). Both freeze drying and spray drying methods produced aquafaba powders that would be preferable for home and industrial use because of their attractive appearance and good water solubility. However, freeze drying is not typically preferred on an industrial scale because of the high capital cost of equipment and the requirement for large amounts of energy.

The amount of water removed and the drying time for different drying methods were calculated and are reported in Table 2. Spray drying removed the largest amount of water (95.0 ± 0.03 g) in the shortest amount of time (0.287 ± 0.001 h) compared with other methods.

The yields of dried aquafaba obtained by different drying methods are presented in Table 2. Rotovap drying provided the highest aquafaba yield by weight (8.78 ± 0.09%) (*p* > 0.05), but the final product had gel-like properties, which could be attributed to Maillard and caramelization reactions caused by the high temperature (50 °C) used in this drying method. The yield of aquafaba dried by vacuum drying was 7.37 ± 0.01%, followed by oven drying (7.22 ± 0.06%) and freeze drying (7.06 ± 0.04%). Spray-dried aquafaba had the lowest yield (5.01 ± 0.03%) (*p* > 0.05) because of sample loss in the lab-scale spray dryer. Some parts of the resulting powder were not properly recovered from the spray dryer because it adhered to the spray dryer parts. These losses would become negligible in a commercial spray dryer.

**Figure 3:**
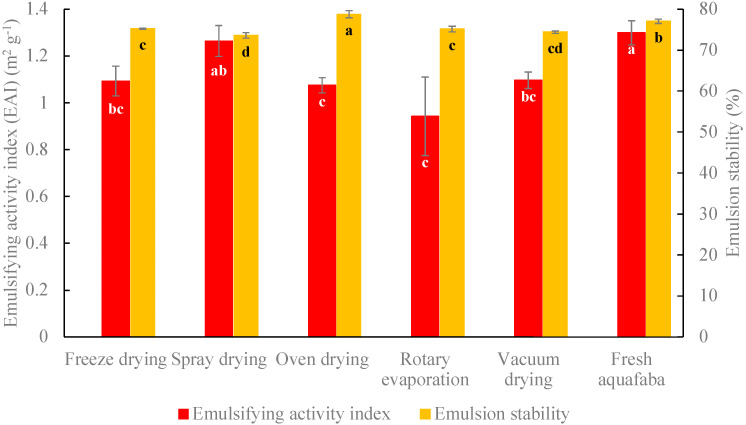
Emulsifying activity index and emulsion stability of rehydrated aquafaba dried by different drying methods. Different letters (a–d) refer to significant differences among different drying methods according to Tukey’s test (*p* < 0.05).

The dried aquafaba was rehydrated using distilled water to obtain the same concentration of solid materials as fresh aquafaba [16] and mixed with canola oil to make emulsions. Figure 3 shows the emulsion properties of rehydrated aquafaba dried by different drying methods. Spray-dried aquafaba demonstrated comparable EAI (1.26 ± 0.07 m^2^ g^−1^) to freshly prepared aquafaba (1.30 ± 0.05 m^2^ g^−1^) (*p* > 0.05). There were no significant differences between the EAIs of spray-dried (1.30 ± 0.05 m^2^ g^−1^), freeze-dried (1.09 ± 0.06 m^2^ g^−1^), and vacuum-dried samples (1.10 ± 0.04 m^2^ g^−1^) (*p* > 0.05). Oven-dried (1.08 ± 0.03 m^2^ g^−1^) and Rotovap dried (0.942 ± 0.168 m^2^ g^−1^) aquafaba samples showed significantly lower EAIs compared with spray-dried and freshly prepared aquafaba.

Oven-dried aquafaba produced emulsions with the highest ES (78.8 ± 0.9%) and demonstrated slightly higher stability compared with fresh aquafaba emulsion (77.1 ± 0.5%) (*p* > 0.05) [16]. The formation of covalent conjugates between proteins and polysaccharides during drying for more than 12 h at 80 °C because of oxidation and thermal-induced reactions (Maillard and caramelization) of aquafaba components (e.g., polysaccharide and protein) may have contributed to higher emulsion stability [7]. However, there was evidence of unsatisfactory browning in oven-dried samples. The ES of freeze-dried (75.2 ± 0.1%), Rotovap dried (75.1 ± 0.7%) and vacuum-dried (74.4 ± 0.3%) samples did not differ significantly. Spray-dried and vacuum-dried aquafaba samples had similar ES, but the former showed a slightly lower ES (73.6 ± 0.7%) compared with other dried samples.

Freeze drying and spray drying methods were selected for further experiments on the basis of the yield of aquafaba powder, solubility in water, EAI, and ES.

### 3.3. Aquafaba Water Holding Capacity and Oil Absorption Capacity

Freeze-dried powder of aquafaba prepared under condition B demonstrated higher WHC (4.36 *±* 0.20 g/g) and OAC (4.6 *±* 0.26 g/g) than spray-dried aquafaba (WHC, 1.92 *±* 0.09 g/g; OAC, 1.98 *±* 0.12 g/g) (*p* > 0.05) (Table 3). Interestingly, Damian et al. [12] observed significantly lower WHC (1.5 g/g) and OAC (3.2 g/g) of freeze-dried chickpea aquafaba. Two main factors affecting these contradictory observations include various soaking and cooking conditions and differences in aquafaba composition and concentration. As the cooking time progresses, protein denaturation occurs, resulting in hydrophobic molecular regions becoming exposed and thus increasing the oil binding capacity, thereby changing its OAC and WAC properties. In addition, cooking under pressure has been shown to cause protein dissociation, exposing more water/oil-binding sites and increasing both WAC and OAC [24]. Comparatively, in Damian et al.’s [12] study, chickpea seed was boiled in water for 90 min, compared with 30 min in our study. Alsalman et al. [19] indicated that increasing cooking time from 15 to 60 min significantly reduced aquafaba WHC (from 2.4 g/g to 1.6 g/g), supporting the improved WHC observed with this shorter cooking time. In addition, they observed that aquafaba OAC increased with longer cooking time and higher chickpea/water ratio. Although the cooking time in our study was shorter, the higher chickpea/water ratio (1:1 vs. 1:1.75) might play a dominant role in increasing OAC.

### 3.4. Mayonnaise and Analog Stability during Cold Storage

The stability of mayonnaise and analogs was evaluated by studying their microstructure and particle size distribution. The emulsion and heating stability are represented in Table 4. Freshly prepared analogs had 15% lower emulsion stability than egg yolk mayonnaise. The stability of egg yolk mayonnaise remained stable after 28 days of storage, while no significant differences in emulsion stability were observed for either analog A or B (73–85 %) stored up to 21 days, after which the emulsion stability of mayonnaise analog B decreased to 56%.

Analog sample A, made from freeze-dried powder, exhibited similar heating stability compared with analog B (from spray-dried powder) on day 0, day 14, and day 21, and higher heating stability on day 28 (*p* > 0.05). The heating stability of mayonnaise analogs was higher compared with egg yolk mayonnaise. Aquafaba contains heat-stable proteins [17], which might contribute to heating stability. These results are comparable to the emulsion stability and heating stability of other egg-free mayonnaises made from both mono- and diglycerides emulsifier (MDG) and guar gum (GG)/xanthan gum (XG) or a mixture of MDG, GG, and XG [4].

To explain the previous results, the long-term stability of mayonnaise and our analogs were also evaluated in terms of pH and colour. The analog samples were acidic (pH ranging from 3.74 to 4.66) compared with egg yolk mayonnaise, and the pH of all samples remained stable up to 28 days (Table 4). The colour profiles of aquafaba mayonnaise analogs were similar, regardless of the drying method; however, a difference was observed between mayonnaise and the analogs (∆*E*_1_* > 3) [18]. Samples of freshly prepared mayonnaise analog A and B had lower L* (87.6, 85.6 vs. 90.6) and *Ch* values (13.0, 14.9 vs. 20.9) (*p* < 0.05) when compared with mayonnaise C, suggesting a darker appearance and a lower colour intensity. Previous research revealed that emulsion colour can change from gray to an increasingly bright white colour with decreasing droplet size, likely due to an increase in light scattering [25,26,27]. This was further confirmed by the larger droplet sizes observed in analog samples A and B. Comparatively, mayonnaise (C) had the highest b* value because of the higher content of pigments in the egg yolk. Nonetheless, ∆*E*_2_* between freshly prepared mayonnaise and analog, along with those that were stored, were not significantly different, indicating that samples A, B, and C all remained stable during storage. Altogether, the colour differences between mayonnaise and analog (∆*E*_1_*) and colour change after storage (∆*E*_2_*) were similar to those of aquafaba mayonnaise analogs with three different aquafaba-to-oil ratios in previous studies [8,18].

### 3.5. Mayonnaise and Analog Microstructure

**Figure 4:**
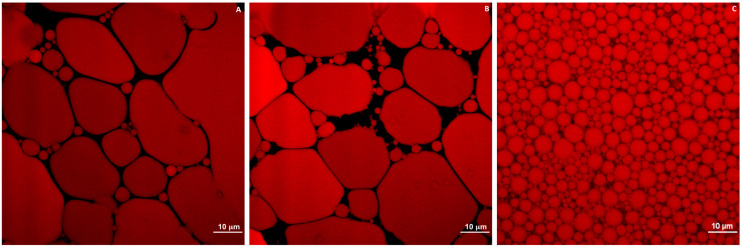
Confocal laser scanning micrographs of mayonnaise and analog prepared with (**A**) freeze-dried aquafaba, (**B**) spray-dried aquafaba, and (**C**) egg yolk. All images were captured at a working magnification of 600× with a 5 times digital zoom. Oil phase was stained with 0.01 wt.% Nile red. Scale bars represent 10 µm.

Confocal laser scanning micrographs of freshly prepared mayonnaise, C, and analogs, A and B, are illustrated in Figure 4. The microstructures of analogs made with both freeze-dried and spray-dried aquafaba were similar, although the interspace voids of analog B (Figure 4B) were slightly larger than in the case of analog A (Figure 4A). The droplets of these aquafaba mayonnaise analog samples were densely packed and revealed polydisperse (oil droplets of different sizes) features. Aquafaba mayonnaise analogs consisted of a large fraction of irregular elliptic oil droplets with greater diameters and a small fraction of spherical oil droplets with smaller diameters. Mayonnaise C (Figure 4C) consisted of finely dispersed, evenly distributed, and significantly smaller spherical oil droplets. It is possible that the large oil droplets became distorted from spherical shape because of the effect of high oil content (80%) and close packing [8]. Moreover, the tightly packed droplets may have also contributed to the stability of the mayonnaise structure [28]. Additionally, coalescence was observed in analog sample A (Figure 4A). Because of the high fat content in mayonnaise and analogs, coalescence is a primary concern for emulsion stability, which is the result of oil droplet convergence [29]. The most effective way to limit coalescence is to generate strong repulsive forces between droplets [4].

The results observed in this study partially contrast with previous research, in which finely diffused spherical oil drops in aquafaba mayonnaise analog were comparable to the microstructure of traditional mayonnaise [8]. Furthermore, Raikos et al. (2020) [8] obtained aquafaba from commercial cans of chickpeas, and mayonnaise analogs were prepared using a homogeniser rather than a hand mixer. The microstructure of mayonnaise and its analogs can be determined by different parameters, including the emulsifying and stabilizing agent types and their concentration, the size of the droplets, oil types and concentrations in mayonnaise and mayonnaise analog formulations, and production process methods [25,30]. Mustafa et al. (2018) [31] showed that aquafaba requires more mixing time to decrease the particle size and obtain functional properties comparable to egg-based foam and emulsion. Therefore, we predict that prolonging the mixing time and applying high-pressure homogenization could help to obtain an aquafaba-based mayonnaise analog with smaller droplet sizes and more stability during storage.

### 3.6. Mayonnaise and Analog Droplet Size Distribution

**Figure 5:**
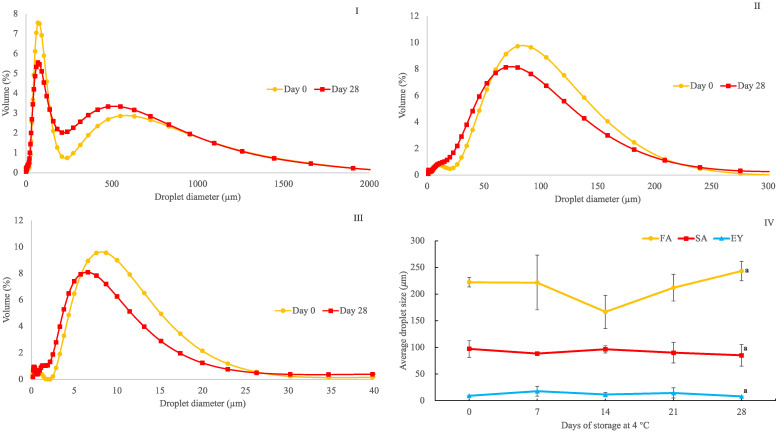
Droplet size distribution of mayonnaise and mayonnaise analog made with freeze-dried aquafaba (**I**), spray-dried aquafaba (**II**) and egg yolk (**III**); and volume average mean droplet diameter (*d*_43_, μm) (**IV**) of mayonnaise and mayonnaise analog as a function of time. The letter (a) refers to no significant difference among storage times according to Tukey’s test (*p* < 0.05).

The variation in the oil droplet size distribution of all mayonnaise and mayonnaise analog samples during storage at 4 °C for 28 days is presented in Figure 5 (I, II, and III). The size distribution for mayonnaise C was 3–15 µm (80% distribution), without significant changes over 28 days. The droplets in mayonnaise analog B were larger and more broadly distributed (20–180 µm) than in mayonnaise C. The analog sample A demonstrated a bimodal droplet distribution with the largest droplet size and range (33–750 µm). No noticeable changes in the droplet size distribution were observed after storing mayonnaise and analogs at 4 °C for 28 days, confirming excellent stability in all samples. Previous work demonstrated differences in droplet size distribution depending on the homogenization qualifications, oil/aqueous phase composition, ingredient viscosity, and emulsion concentration and type [9]. In this study, only the type of emulsifiers was changed. The particle size range of analog B was similar to those in previous reports by Liu et al. (2007) and Di Mattia et al. (2015) [32,33] but was also markedly higher than those obtained by Laca et al. (2010), Nikzade et al. (2012), and Raikos et al. (2020) [4,8,34].

The average droplet size (*d*_43_) of analog B, 97.3 ± 16 µm, was smaller than that of analog A, 222 ± 9 µm, for all times in the 28-day trial (*p* < 0.05). However, both analogs A and B had significantly larger particle sizes than mayonnaise C (9.02 ± 1 µm). This was also visually evident in the CLSM, suggesting a lower capacity of aquafaba to emulsify and stabilize the mixture compared with egg yolk-based mayonnaise. This result indicates a wider variety of aquafaba mayonnaise analog microstructures depending on the emulsifier condition and the composition [32]. Droplet size is a crucial parameter in mayonnaise and analog evaluation, as it affects rheology, stability, storage life, texture, and taste [35]. In general, oil droplets with smaller particle sizes help in decreasing the movement of droplets and inhibiting coalescence, sedimentation, and other instabilities within the emulsion system, thereby increasing mayonnaise and analog viscosity and stability [9].

The difference in droplet size for analogs A and B suggests an effect of the drying process on aquafaba emulsifying properties. Since the dry matter of aquafaba consists primarily of carbohydrates and proteins, some chemical reactions (e.g., the Maillard reaction) can occur under high-temperature conditions during the spray drying process, promoting protein glycation and the formation of covalent conjugates between proteins and polysaccharides. These interactions can influence aquafaba emulsifying properties, causing a difference between the droplet sizes of analogs A and B [36,37,38,39]. Xu and Zhao [38] stated that these protein–polysaccharide conjugates can be easily absorbed in oil-water interfaces to form a thick and stable film and improve colloidal stability.

Different emulsifying components of aquafaba and egg yolk can explain why aquafaba mayonnaise analogs exhibited larger droplet sizes. Stantiall et al. (2018) reported that aquafaba dry matter mainly consisted of insoluble polysaccharides (46%), water-soluble carbohydrates (24%), and protein (19%) [13]. Polysaccharides have remarkable WHC and thickening properties conferred from their hydrophilicity and high molecular weight. These properties can provide high viscosity of the aqueous phase and restrain the formation of fine, evenly distributed droplets in vegan mayonnaise analogs. In addition, because of the high concentration and high molecular weight of insoluble polysaccharides, a thick gel layer can be produced around the oil droplets to further enlarge their size [9]. Comparatively, the size of emulsifier molecules in egg is much smaller (mainly lecithin, lipoprotein, livetin, and phosvitin) [34,40]. Generally, small molecular size emulsifiers can generate small fat droplets and promote superior stability in an emulsion system [41,42].

## 4. Conclusions

The impacts of different cooking and drying methods on aquafaba emulsion capacity and stability were investigated. Dried aquafaba powder was used as an emulsifier in a vegan mayonnaise analog, and the physicochemical properties and stability were compared to traditional mayonnaise made from egg yolk. Aquafaba prepared by soaking chickpea seed in 4 °C water for 16 h and cooking for 30 min achieved the highest emulsion capacity and stability. When compared with other drying methods, freeze drying and spray drying produced powdered aquafaba with superior emulsion properties. Spray drying displayed a shorter drying time. Both freeze-dried and spray-dried aquafaba can be used to produce vegan mayonnaise analogs with comparable pH, colour, and stability to egg yolk mayonnaise. However, the droplet size in mayonnaise analogs made from freeze-dried and spray-dried aquafaba were much larger than in egg-yolk mayonnaise. This reduced the long-term stability compared with egg yolk mayonnaise. This study lays the foundation for commercial production of aquafaba powder for long-term storage and transportation and demonstrates the use of dried aquafaba as an egg replacement and emulsifier in the formulation of vegan mayonnaise analogs. Further studies will evaluate the effect of processing methods and investigate the effects of dried aquafaba concentration on the microstructure and stability of aquafaba mayonnaise analogs.

## Figures and Tables

**Table 1:** Aquafaba production conditions.

Condition	A	B	C	D	E
Soaking time (h)	16	16	16	1	1
Soaking temperature (°C)	4	4	4	85	85
Soaking water additives (*w*/*w*)	NA	NA	NA	NA	0.2% NaHCO_3_
Cooking time (min)	20	30	60	30	20

NA—no additives.

**Table 2:** Water removed, drying time, and dried aquafaba yield of different drying methods for 100 g fresh aquafaba and water added to rehydrate aquafaba.

Drying Methods	Water Removed (g)	Drying Time (h)	Dried Aquafaba Yield (g/100 g Fresh Aquafaba)	Water Added (g/10 g Dried Aquafaba)
Spray drying	95.0 ± 0.03 ^a^	0.287 ± 0.001 ^d^	5.01 ± 0.03 ^e^	190
Freeze drying	92.9 ± 0.04 ^b^	129 ± 5 ^a^	7.06 ± 0.04 ^d^	132
Oven drying	92.8 ± 0.06 ^c^	29.0 ± 2.1 ^c^	7.22 ± 0.06 ^c^	129
Rotovap drying	91.2 ± 0.09 ^e^	3.22 ± 0.08 ^d^	8.78 ± 0.09 ^a^	104
Vacuum drying	92.6 ± 0.01 ^d^	45.6 ± 1.5 ^b^	7.37 ± 0.01 ^b^	126

Data are expressed as means ± standard deviation (*n* = 3). Different letters (^a–e^) refer to significant differences among different drying methods according to Tukey’s test (*p* < 0.05).

**Table 3:** Physicochemical properties of freeze-dried and spray-dried aquafaba.

Dried Aquafaba	Freeze-Dried Aquafaba	Spray-Dried Aquafaba
Moisture content (%)	5.17 ± 0.21 ^a^	2.50 ± 0.01 ^b^
WHC (g/g)	4.36 ± 0.20 ^a^	1.92 ± 0.09 ^b^
OAC (g/g)	4.64 ± 0.26 ^a^	1.98 ± 0.12 ^b^

WHC, water holding capacity; OAC, oil absorption capacity. Data are expressed as means ± standard deviation (*n* = 3). Different letters (^a,b^) refer to significant differences according to Tukey’s test (*p* < 0.05).

**Table 4:** Physicochemical properties of mayonnaise and analogs over 28 days of storage at 4 °C.

Mayonnaise and Analog	A	B	C
Emulsifier	Freeze-Dried Aquafaba (FA)	Spray-Dried Aquafaba (SA)	Egg Yolk (EY)
Day 0			
pH	3.99 ± 0.17 ^Ab^	3.74 ± 0.10 ^Bb^	4.66 ± 0.07 ^Aa^
L*	87.6 ± 0.03 ^Ab^	85.6 ± 0.04 ^Ac^	90.6 ± 0.1 ^Aa^
a*	−2.31 ± 0.02 ^Bb^	−2.17 ± 0.01 ^Bb^	−1.85 ± 0.02 ^Ac^
b*	12.8 ± 0.02 ^Bc^	14.8 ± 0.05 ^Bb^	20.8 ± 0.09 ^Ba^
Ch	13.0 ± 0.03 ^Bc^	14.9 ± 0.05 ^Bb^	20.9 ± 0.09 ^Ba^
∆*E*_1_*	8.56 ± 0.13 ^a^	7.82 ± 0.15 ^b^	
Emulsion stability	85.0 ± 3.2 ^Ab^	84.6 ± 2.0 ^Ab^	100 ± 0 ^Aa^
Heating stability	68.3 ± 5.0 ^Aa^	62.8 ± 1.7 ^Bab^	59.4 ± 1.0 ^ABb^
Day 7			
pH	4.05 ± 0.03 ^Ab^	4.00 ± 0.10 ^Ab^	4.50 ± 0.06 ^Ba^
Emulsion stability	83.4 ± 4.3 ^ABb^	83.6 ± 1.6 ^Ab^	100 ± 0 ^Aa^
Heating stability	70.2 ± 2.4 ^Ab^	76.2 ± 0.8 ^Aa^	61.6 ± 1.3 ^Ac^
Day 14			
pH	4.07 ± 0.02 ^Ab^	4.04 ± 0.07 ^Ab^	4.42 ± 0.01 ^Ba^
Emulsion stability	82.9 ± 4.2 ^ABb^	76.2 ± 3.7 ^ABb^	100 ± 0 ^Aa^
Heating stability	62.8 ± 8.0 ^Aa^	58.7 ± 7.1 ^Ba^	48.8 ± 1.4 ^BCa^
Day 21			
pH	4.02 ± 0.03 ^Ab^	3.98 ± 0.05 ^Ab^	4.40 ± 0.01 ^Ba^
Emulsion stability	75.4 ± 1.4 ^Bb^	73.3 ± 2.3 ^Bb^	100 ± 0 ^Aa^
Heating stability	66.5 ± 5.2 ^Aa^	57.6 ± 1.4 ^Bab^	48.3 ± 8.1 ^Cb^
Day 28			
pH	4.05 ± 0.09 ^Ab^	3.94 ± 0.03 ^Ab^	4.46 ± 0.02 ^Ba^
L*	82.4 ± 0.2 ^Bb^	81.7 ± 0.01 ^Bc^	88.2 ± 0.09 ^Ba^
a*	−2.67 ± 0.02 ^Aa^	−2.33 ± 0.01 ^Ab^	−0.587 ± 0.040 ^Bc^
b*	13.7 ± 0.08 ^Ac^	17.0 ± 0.04 ^Ab^	25.4 ± 0.1 ^Aa^
Ch	14.0 ± 0.08 ^Ac^	17.1 ± 0.04 ^Ab^	25.4 ± 0.1 ^Aa^
∆*E*_1_*	13.2 ± 0.3 ^a^	10.8 ± 0.2 ^b^	
∆*E*_2_*	5.30 ± 0.23 ^a^	4.50 ± 0.04 ^b^	5.35 ± 0.08 ^a^
Emulsion stability	79.7 ± 2.7 ^ABb^	55.9 ± 5.8 ^Cc^	100 ± 0 ^Aa^
Heating stability	66.4 ± 5.7 ^Aa^	54.6 ± 3.9 ^Bb^	48.5 ± 3.8 ^BCb^

Ch, chroma; ∆*E*_1_*, colour difference from the control; ∆*E*_2_*, colour difference of mayonnaise and analog samples during cold storage. Data are expressed as means ± standard deviation (*n* = 3). Different letters (^a–c^) refer to significant differences according to Tukey’s test (*p* < 0.05). Lower case letters show significant differences among different emulsifiers. Capital letters show significant differences among storage time.

## Data Availability

The datasets generated for this study are available on request to the corresponding author.
